# Correction to ‘Full-length direct RNA sequencing reveals extensive remodeling of RNA expression, processing and modification in aging *Caenorhabditis elegans*’

**DOI:** 10.1093/nar/gkag603

**Published:** 2026-06-04

This is a correction to: Erin C Schiksnis, Ian A Nicastro, Amy E Pasquinelli, Full-length direct RNA sequencing reveals extensive remodeling of RNA expression, processing and modification in aging *Caenorhabditis elegans, Nucleic Acids Research*, Volume 52, Issue 22, 11 December 2024, Pages 13896–13913, https://doi.org/10.1093/nar/gkae1064

In the originally published version of this manuscript, Supplementary Table S1 contains a duplicated sheet that was incorrectly labelled as a separate dataset.

Specifically, in the supplementary file entitled “01_DESeq_WS279_all_days_vDay1”, the sheet ”DESeq_day1_day15_nanopore" is an inadvertent duplicate of the sheet “DESeq_day1_day10_nanopore”.

This has been corrected and the updated supplementary file is included in the Supplementary data section of this correction notice.

These details have been corrected only in this correction notice to preserve the published version of record.

## Supplementary Material

gkag603_Supplemental_File

