# Supplementary material for: Full-length direct RNA sequencing reveals extensive remodeling of RNA expression, processing and modification in aging Caenorhabditis elegans
Source: Nucleic Acids Res. 2024 Nov 18;52(22):13896–913. doi: 10.1093/nar/gkae1064 (PMC11662692; doi:10.1093/nar/gkae1064)
Supplement: gkae1064_Supplemental_Files [file gkae1064_supplemental_files.zip › Schiksnis_NAR_DocS1_revised_FINAL.pdf]

Supplemental Information

Full-length direct RNA sequencing reveals extensive remodeling of RNA expression, processing and modification in aging *Caenorhabditis elegans*

Erin C. Schiksnis, Ian A. Nicastro, and Amy E. Pasquinelli

<u>In this PDF:</u>
Document S1. Figures S1-S7

<u>In separate EXCEL files:</u>
Table S1. Illumina and Nanopore RNA-seq DESeq2 results from days 2, 3, 4, 5, 7, 10, and 15 vs. day 1.

Table S2. Number of Nanopore DRS reads per replicate remaining after each filtering step (mapping, transcription start site filtering, and poly(A) filtering).

Table S3. Genomic coordinates for all detected Nanopore DRS full-length isoforms and 3'UTRs and the number of filtered reads for each isoform per replicate. Novel isoforms not in the WS279 WormBase annotation are labeled as productive if they span in frame annotated start and stop codons or unproductive if they do not span in frame annotated start and stop codons. 3'UTR isoforms are labeled as novel or annotated as compared to WS279.

Table S4. Alternative exon inclusion events with a significant ($P<0.05$) change in relative abundance (PSI) in days 2, 3, 4, 5, 7, 10, or 15 compared to day 1.

Table S5. Percent of reads mapping to introns or intergenic regions and percent of splice junction reads that do not match WS279 WormBase annotations in young (days 1, 2, 3) and old (days 7, 10, 15) animals.

Table S6. Median, mean, minimum, and maximum poly(A) tail lengths for isoforms with full-length support.

Table S7. High confidence A-to-G edit site coordinates in BED format, features that overlap with A-to-G sites, and predicted codon changes resulting from editing.

Table S8. High confidence pseudouridine edit site coordinates in BED format and overlapping features.

Table S9. Annotated aging genes with novel features from this study indicated.

Supplementary Figures

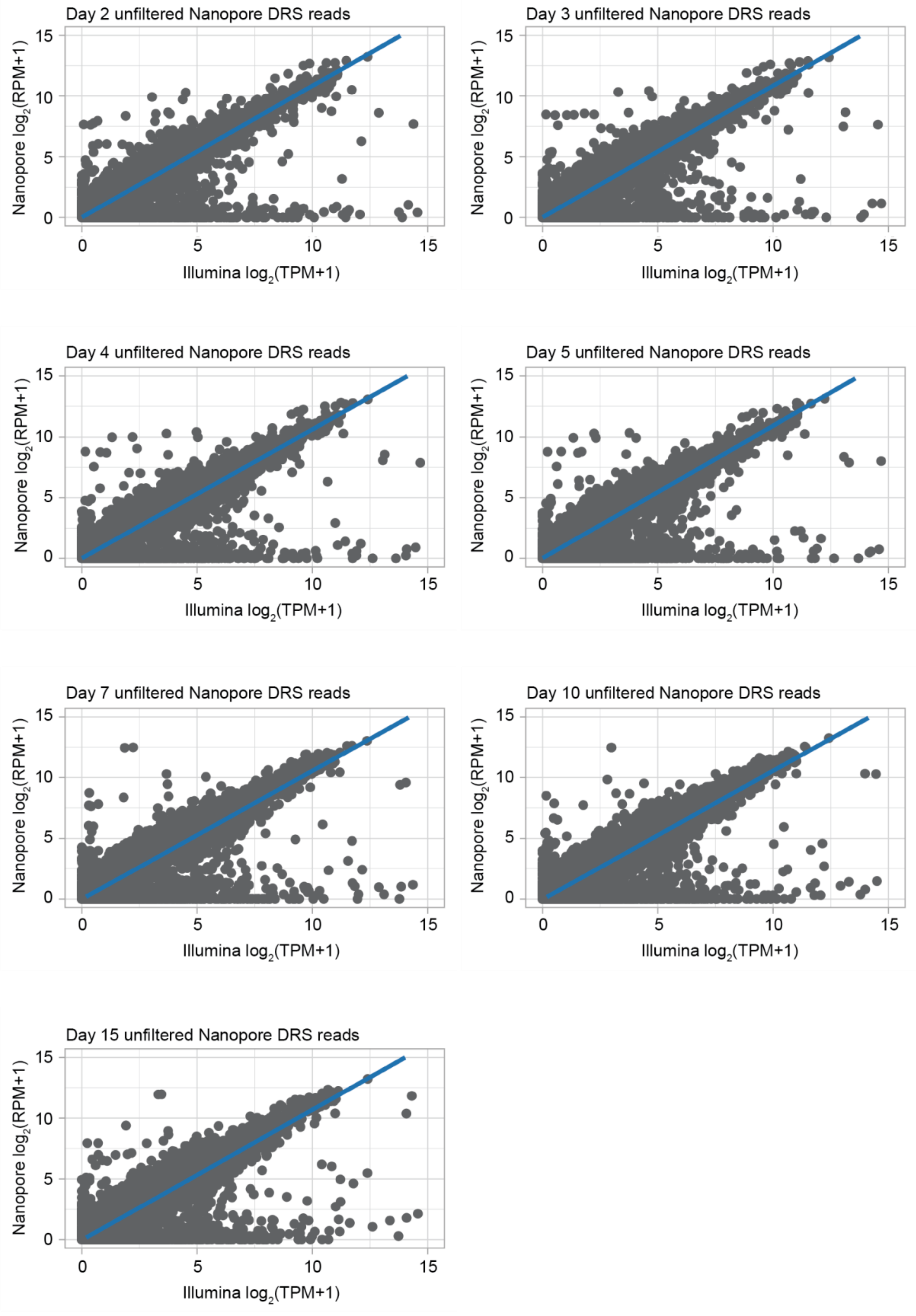

Figure S1, related to Figure 1. Correlations between Nanopore DRS and Illumina RNA-seq normalized gene expression. Scatterplots comparing mean normalized expression of individual genes across three independent biological replicates between Nanopore DRS ($\log_2$ TPM + 1) and Illumina RNA-seq ($\log_2$ RPM + 1) at each time point.

A **B**

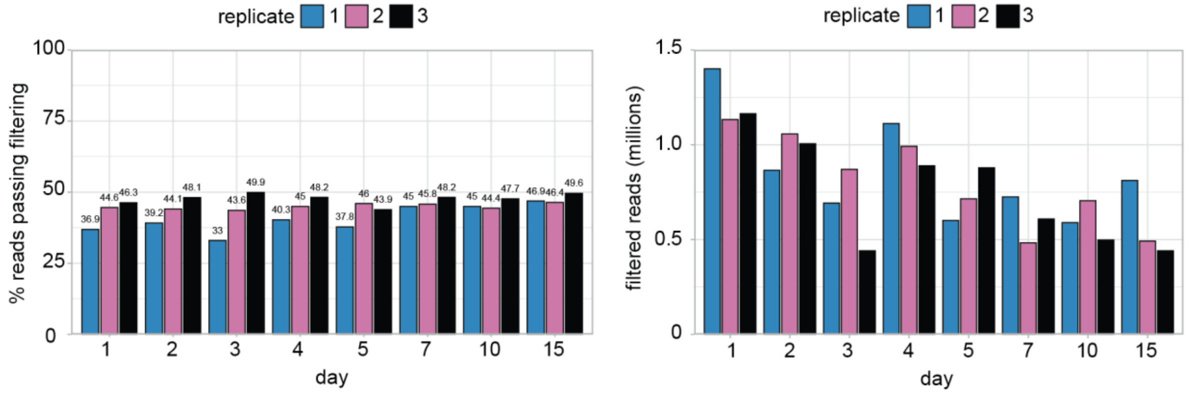

C

Day	Nanopore (full-length only) and Illumina correlation (R^2)
1	0.51
2	0.53
3	0.52
4	0.54
5	0.54
7	0.50
10	0.48
15	0.48

D

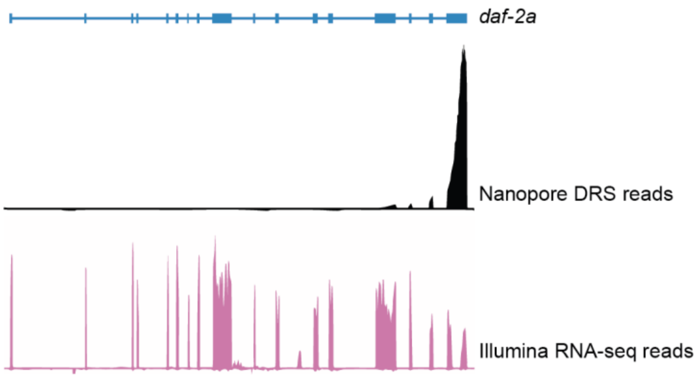

Figure S2, related to Figure 2. Read filtering for Nanopore DRS. A. Percent of reads that remain after Nanopore filtering pipeline at each time point. **B.** Number of reads that remain after Nanopore filtering pipeline at each time point. **C.** Table showing the R-squared value for mean normalized expression for filtered Nanopore DRS ($\log_2$ TPM + 1) and Illumina RNA-seq ($\log_2$ RPM + 1) of individual genes across three independent biological replicates at each time point. **D.** Browser track image of *daf-2a* showing unfiltered Nanopore DRS and Illumina RNA-seq read density.

A

Gene fusion	Total counts	Break 1	Break 2
C18H9.6:*clec-173*	728	II:6701465 (-)	IV:3266420 (-)
T28F3.8:*clec-84*	293	IV:17307321 (-)	IV:2845291 (+)
Y51H4A.24:*clec-84*	62	IV:16739384 (-)	IV:2845291 (+)
eri-6:*eri-7*	57	I:4458645 (+)	I:4456339 (-)
clec-84:K08D10.14	66	IV:2845291 (+)	I:4456339 (-)

B

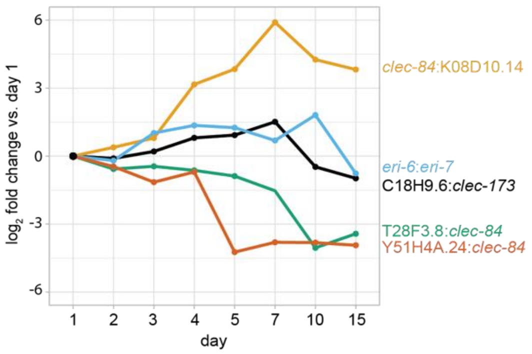

C

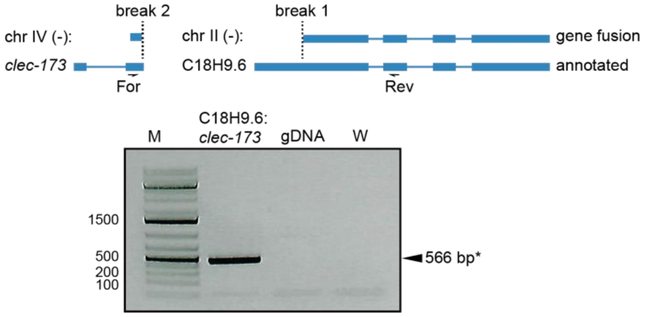

Figure S3, related to Figure 2. Detection of gene fusion isoforms at adult time points. A. Table of gene fusions detected with LongGF. Break points indicate the genomic coordinates where two genes are joined. **B.** Line plot showing the relative expression normalized to day 1 of LongGF fusion genes. **C.** RT-PCR validation of the C18H9.6:*clec-173* LongGF fusion isoform. Agarose gel electrophoresis (*bottom*) with expected fragment size denoted, including genomic DNA and water controls. Schematic representations of gene fusion (*top*) showing break points and forward and reverse PCR primers used for amplification of cDNA.

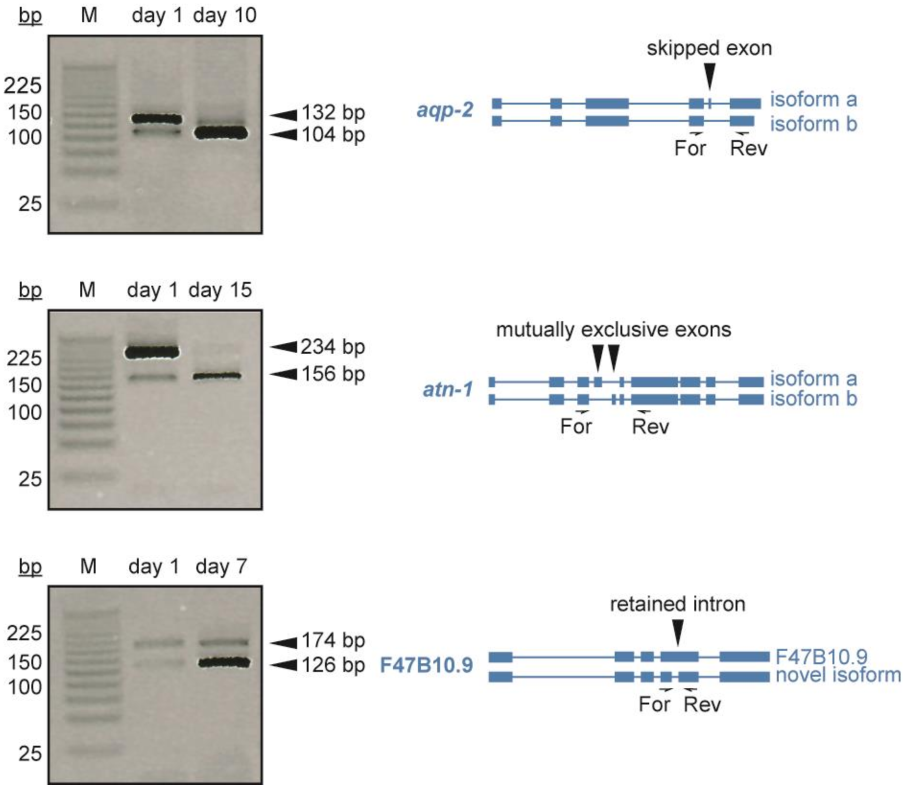

Figure S4, related to Figure 3. Validation of alternative exon inclusion events in aging. Semi-quantitative RT-PCR at indicated time points using primers flanking alternative exon inclusion events identified with SUPPA2. Agarose gel electrophoresis (*left*) with expected fragment sizes denoted. Schematic representations show alternatively spliced isoforms (*right*) and forward and reverse PCR primers used for amplification of cDNA.

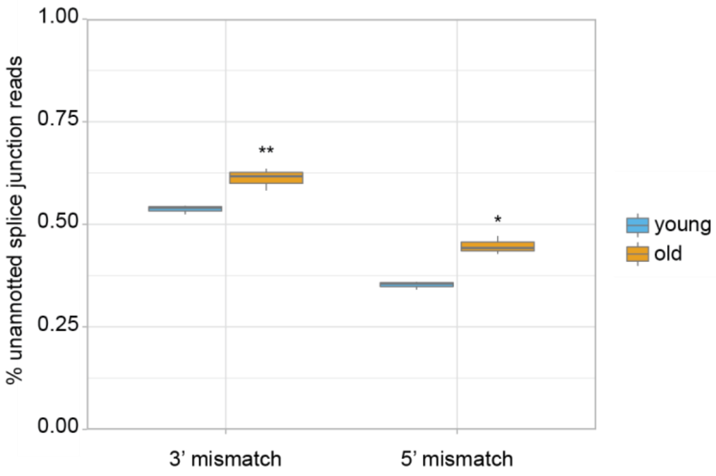

Figure S5, related to Figure 4. Percent of splice junction reads using unannotated 5' or 3' splice sites. Boxplot showing the percent of splice junction reads that do not match WS279 annotated 5' splice sites (***P*=0.004, *t*-test, two-sided after probit transformation) or 3' splice sites (**P*=0.022, *t*-test, two-sided after probit transformation).

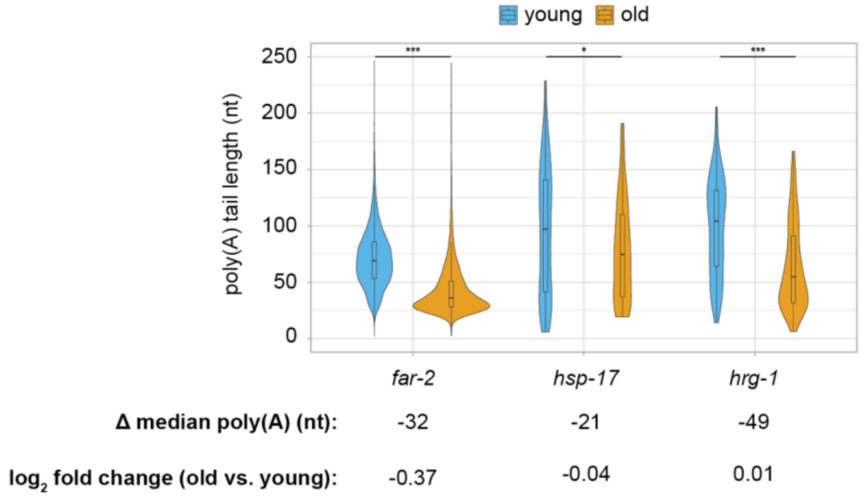

Figure S6, related to Figure 4. Genes with large changes to poly(A) tail lengths in aging. Violin plot with inlaid boxplot of poly(A) tail length distribution for reads assigned to indicated genes. Changes to media poly(A) tail lengths and expression in old compared to young animals are indicated. ***P<0.001, *P<0.05 (Kolmogorov-Smirnov test) between young and old animals.

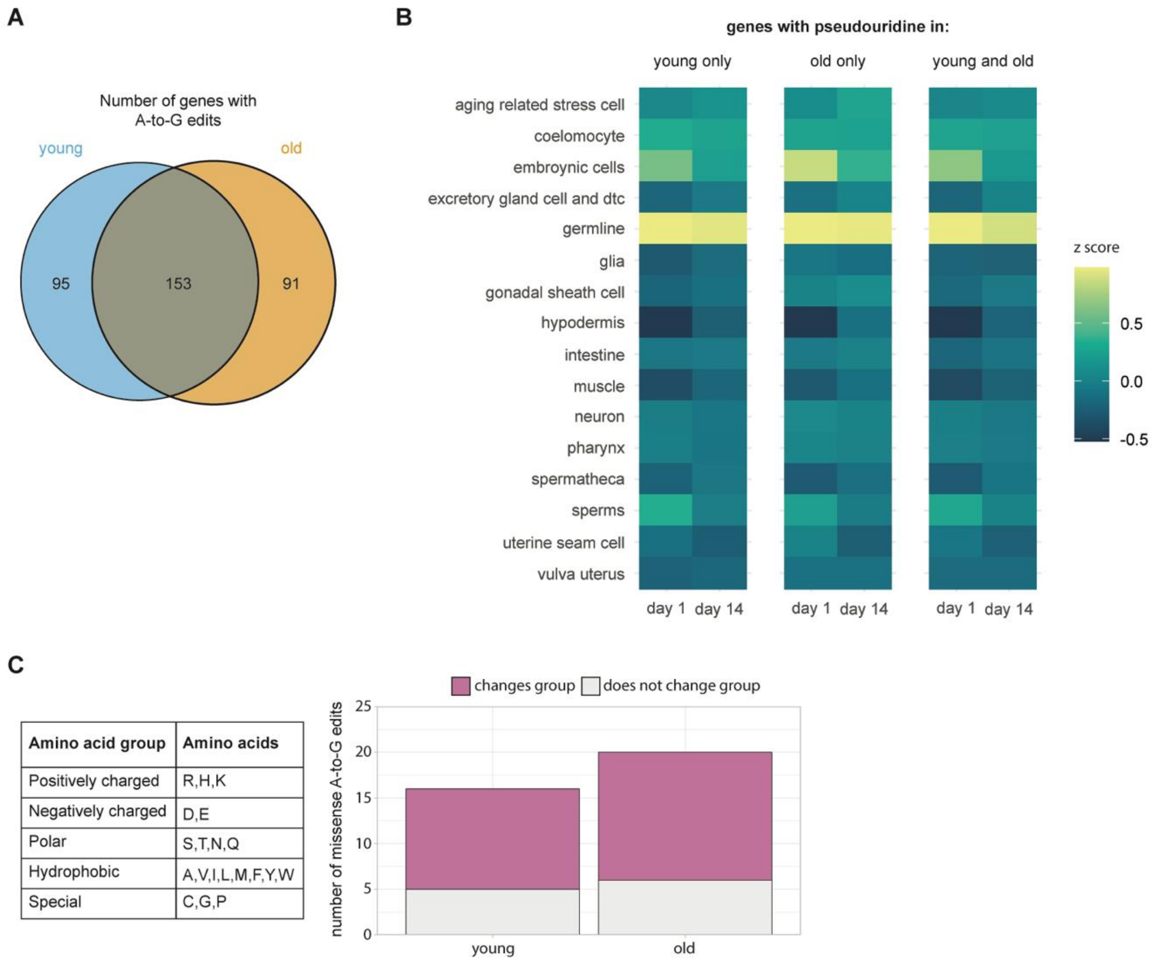

Amino acid group	Amino acids
Positively charged	R,H,K
Negatively charged	D,E
Polar	S,T,N,Q
Hydrophobic	A,V,I,L,M,F,Y,W
Special	C,G,P

Figure S7, related to Figure 5. Examination of A-to-I editing in aging. A. Venn diagram with the number of genes with A-to-G edits in young and old animals. **B.** Heatmaps of tissue enrichment scores of genes with inosine detected at young time points, old time points, or young and old time points. **C.** Table with amino acid group characterizations based on chemical properties (*left*) and stacked bar plot showing the number of predicted missense A-to-G edits that result in a change to the encoded amino acid group (*right*).

A

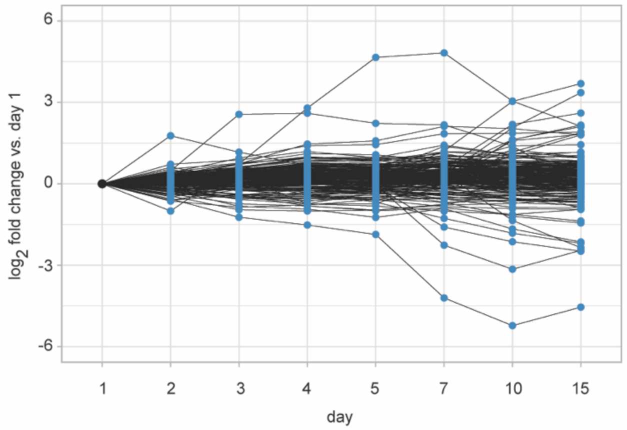

B

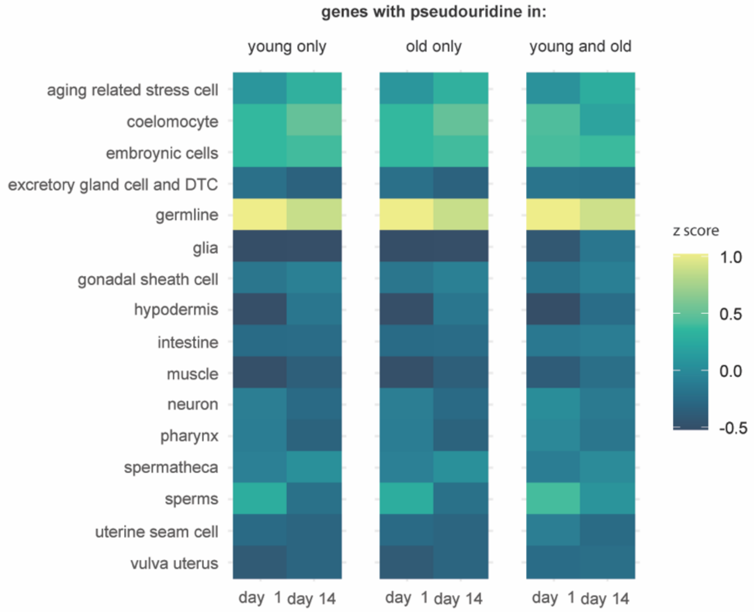

C

Gene	Human homolog	Target(s) of human homolog
pus-1	Pus1	tRNA
tag-124	Pus3	tRNA, mRNA
Y43B11AR.3	TruB2	tRNA, mt-mRNA
K07E8.7	RpusD2	mRNA
ZK287.7	RpusD3, RPusD4	mt-rRNA, tRNA
B0024.11	Pus7	tRNA, mRNA
Y48C3A.20	Pus10	tRNA, rRNA, snRNA

D

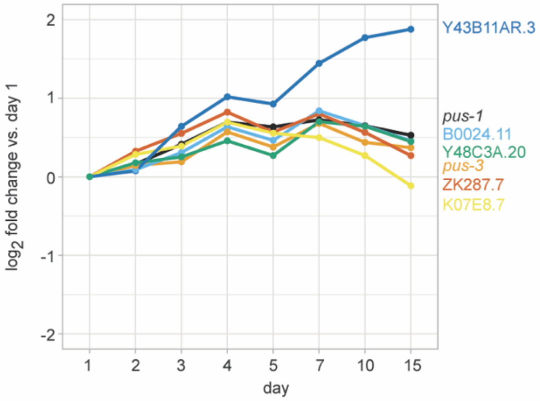

Figure S8, related to Figure 6. Expression of genes with pseudouridine and pseudouridine synthase enzymes. A. Line plot showing the relative expression normalized to day 1 of genes with pseudouridine in old animals but not young. Each blue dot represents an individual gene. **B.** Heatmaps of tissue enrichment scores of genes with pseudouridine detected at young time points, old time points, or young and old time points. of **C.** Table of all predicted *C. elegans* homologs of human pseudouridine synthase enzymes and their RNA targets in human. **D.** Line plot showing the relative expression normalized to day 1 of predicted pseudouridine synthase genes.
